# Therapeutic strategies in epithelial ovarian cancer

**DOI:** 10.1186/1756-9966-31-14

**Published:** 2012-02-13

**Authors:** Ayako Kim, Yutaka Ueda, Tetsuji Naka, Takayuki Enomoto

**Affiliations:** 1 Department of Obstetrics and Gynecology, Osaka University Graduate School of Medicine, 2-2, Yamadaoka, Suita, Osaka 565-0871, Japan; 2 Laboratory for Immune Signal, National Institute of Biomedical Innovation, Osaka, Japan

**Keywords:** Review, ovarian cancer, conventional treatment, novel treatment, clear cell carcinoma, bevacizumab, PARP inhibitor

## Abstract

Ovarian cancer is the most lethal gynecologic malignancy. It appears that the vast majority of what seem to be primary epithelial ovarian and primary peritoneal carcinomas is, in fact, secondary from the fimbria, the most distal part of the fallopian tube.

Treatment of epithelial ovarian cancer is based on the combination of cytoreductive surgery and combination chemotherapy using taxane and platinum. Although clear cell type is categorized in indolent type, it is known to show relatively strong resistance to carboplatin and paclitaxel regimen and thus poor prognosis compared to serous adenocarcinoma, especially in advanced stages. Irinotecan plus cisplatin therapy may effective for the clear cell adenocarcinoma.

The larger expectation for improved prognosis in ovarian carcinoma is related to the use of the new biological agents. One of the most investigated and promising molecular targeted drugs in ovarian cancer is bevacizumab, a monoclonal antibody directed against VEGF. PARP inhibitor is another one. A few recent studies demonstrated positive results of bevacizumab on progression-free survival in ovarian cancer patients, however, investigation of molecular targeting drugs in patients with ovarian cancer are still underway.

## Background

Ovarian cancer is the most lethal gynecologic malignancy. The origin and pathogenesis of epithelial ovarian cancer (EOC) have long been investigated but still poorly understood. Studies have shown that epithelial ovarian cancer is not a single disease but is composed of a diverse group of tumors that can be classified based on distinctive morphologic and molecular genetic features [1].

Treatment of epithelial ovarian cancer (EOC) is based on the combination of surgery and chemotherapy. Over the past three decades, surgical tumor debulking, followed by platinum-based chemotherapy is the standard treatment for advanced ovarian cancer. Although response rates and complete responses in advanced disease are >80% and 40-60%, respectively, after first-line treatment with carboplatin and paclitaxel, most patients will eventually relapse with a median progression-free survival of 18 months [2]. Intraperitoneal chemotherapy possibly improve progression-free and overall survivals (PFS and OS), however, intraperitoneal chemotherapy has not been universally accepted for at least three reasons: toxic effects, intraperitoneal treatment delivery issues and complications [3].

In this review, we first focus on the origin and pathogenesis of EOC, introducing emerging concepts of a unifying theory. Next we look at the history of treatment of EOC as well as novel treatment strategies (e.g. molecular targeted treatment).

### Classification of epithelial ovarian cancer

Kurman et al. have proposed a dualistic model that categorizes various types of epithelial ovarian cancer into two groups designated type I and type II [1,4,5]. Type I tumors are clinically indolent and usually present at a low stage, while type II tumors exhibit papillary, grandular, and solid patterns and are highly aggressive and almost always present in advanced stage (Table 1). Type I tumors include low-grade serous, low-grade endometrioid, clear cell and mucinous carcinomas and type II include high-grade serous, high-grade endometrioid and undifferentiated carcinomas. Malignant mixed mesodermal tumors (carcinosarcomas) are included in the type II category because their epithelial components are identical to the pure type II carcinomas.

**Table 1:** Characteristics of type I and type II tumors

	Type I	Type II
Clinical features	indolent	aggressive

Histological features	low-grade serous	high-grade serous
	low-grade endometrioid	high-grade endometrioid
	clear cell	undifferentiated
	mucinous	carcinosarcoma

Molecular features	K-Ras	TP53CCNE1
	BRAF	
	ERBB2	
	PTEN	
	CTNNB1	
	PIK3CA	

Type I and type II tumors have remarkably different molecular genetic features as well as morphologic differences. For example, high-grade serous carcinoma (type II tumor) is characterized by very frequent *TP53 *mutations (> 80% of cases) and *CCNE1 *(encoding cyclin E1) amplification but rarely has mutations that characterize most type 1 I tumors such as *KRAS, BRAF, ERBB2, PTEN, CTNNB1, and PIK3CA *[6]. In general, type I tumors are genetically more stable than type II tumors and display a distinctive pattern of mutations that occur in specific cell types. Type II tumors which show greater morphologic and molecular homogeneity are genetically unstable and have a very high frequency of *TP53 *mutations. These findings suggest that these two different types of ovarian cancers develop along different molecular pathways.

In terms of origin of ovarian cancer, many of researchers and gynecologic oncologists have traditionally understood that the various different ovarian tumors are all derived from the ovarian surface epithelium (mesothelium) and that subsequent metaplastic changes lead to the development of the different cell types (Table 2). It is well known that serous, endometrioid, clear cell, mucinous and transitional cell (Brenner) carcinomas morphologically resemble the epithelia of the fallopian tube, endometrium, gastrointestinal tract or endocervix and urinary bladder, respectively. The normal epithelial cells of the ovary, however, do not show any resemblance with these tumors. An alternate theory proposes that tumors with a mullerian phenotype (serous, endometrioid and clear cell) are derived from mullerian-type tissue not mesothelium. It has been suggested that they could arise from tissues ovarian epithelial tumors are embryologically derived from the mullerian duct [7]. This mullerian-type tissue (columnar epithelium, often ciliated) forms cysts located in paratubal and paraovarian locations. According to this theory, ovarian tumors develop from these cysts, not the ovarian surface epithelium. As the tumor enlarges, it compresses and eventually obliterates ovarian tissue resulting in an adnexal tumor that appears to have arisen in the ovary.

**Table 2:** Origin of ovarian carcinoma

	Serous	Endometrioid/Clear	Mucinous/Brenner
Traditional theory	ovarian surface epithelium (mesothelim)	ovarian surface epithelium (mesothelim)	ovarian surface epithelium (mesothelim)

Recent theory	fimbria	endometrial tissue (endometriosis)	tubal-mesothelial junction

In summary, it appears that the vast majority of what seem to be primary epithelial ovarian and primary peritoneal carcinomas are, in fact, secondary. Previous data support the view that serous tumors develop from the fimbria, the most distal part of the fallopian tube, endometrioid and clear cell tumors from endometrial tissue passing through the fallopian tube resulting in endometriosis and mucinous and Brenner tumors from transitional-type epithelium located at the tubal-mesothelial junction where the fimbria makes contact to the peritoneum.

Although the data suggesting that epithelial ovarian carcinoma arises in extra-ovarian sites and involves the ovaries secondarily are compelling, low- and high-grade serous carcinomas involve the ovaries and other pelvic and abdominal organs, such as the omentum and mesentery, much more extensively than the fallopian tubes. Similarly, although endometrioid carcinomas develop from endometriosis, which frequently involves multiple sites in the pelvis, these tumors are usually confined to the ovaries. It is likely that the predisposition for growth in the ovary is multifactorial but the precise reasons for this are unknown.

The proposed model by assigning different epithelial ovarian tumors into two categories based on clinical, morphological, and molecular genetic characteristics could serve as a framework for studying ovarian cancer pathogenesis, but this model is not complete and does not resolve all the issues. For example, clear cell carcinoma and mucinous cadenocarcinoma are classified as type I tumors, but unlike the other type I tumors clear cell and mucinous cell types are often high-grade at presentation and show relatively strong resistance to platinum-based chemotherapy. This model does not replace traditional histopathologic classification but can be expected to draw attention to the molecular genetic events that play a role in the tumor progression and can give light on new approaches to early detection and treatment of ovarian cancer.

### Conventional treatment of EOC

#### Early disease: FIGO stage I-II

Due to the lack of effective screening programs, ovarian cancer is diagnosed at an early stage only in about 25% of the cases. In most of these cases surgery is able to cure the disease, and the five-year survival rate for early-stage (stage I or II) ovarian cancer is around 90% [3]. Adjuvant chemotherapy for early stage ovarian cancer is still controversial but some studies have shown its benefit under confined conditions. According to the results of two studies from the International Collaborative Ovarian Neoplasm group and the EORTC, patients with IA or IB FIGO stage, non-clear-cell histology, well-differentiated (G1) tumors, and an "optimal" surgery (performed according to international guidelines, with pelvic and retroperitoneal assessment), appear not to benefit from chemotherapy [8]. Thus, it is commonly believed that, at least in these cases chemotherapy can be probably avoided and patients can be advised to undergo clinical and instrumental follow-up. In all the other (early stage) patients (adjuvant) chemotherapy is indicated [3].

#### Advanced disease: FIGO III-IV

The standard treatment for patients with advanced ovarian cancer is maximal surgical cytoreduction (total abdominal hysterectomy, bilateral salpingo-oophorectomy, pelvic and para-aortic lymphadenectomy and omentectomy) followed by systemic platinum-based chemotherapy and, actually, is reasonable to expect a 5-year survival for 10-30% of women diagnosed with ovarian cancer at stage III or IV [3]. The concept of primary debulking surgery is to diminish the residual tumor burden to a point at which adjuvant therapy will be optimally effective. The percentage of patients with advanced ovarian cancer who can optimally undergo cytoreductive surgery seems to range from 17%-87% [9], depending on the report reviewed. This percentage can largely depend on the experience of the surgeon.

Recently, an interesting randomized control trial on treatment of advanced ovarian cancer was conducted by Vergote et al. [10]. This phase III randomized study compared primary debulking surgery followed by chemotherapy with neoadjuvant chemotherapy followed by interval debulking surgery in patients with advanced ovarian cancer (Table 3). The median overall survival was 29 months in the primary-surgery group and 30 months in the neoadjuvant chemotherapy group and this difference was not statistically significant. Also, n difference was observed in median progression-free survival. These results are thoroughly discussed among the experts in this field; it is believed that upfront maximal cytoreduction is still the standard, although further research should focus on how to select patients that cannot receive optimal cytoreduction and that can benefit from a neoadjuvant strategy. When deciding debulking surgery, we should assess predictive factors with respect to recidual macroscopic disease after debulking surgery which is the strongest independent variable in predicting survival [10].

**Table 3:** Comparison of primary debulking surgery and neoadjuvant chemotherapy

	Primary debulking surgery	Neoadjuvant chemotherapy
Number of patients	336	334

Age: Median (range)	62 (25-86)	63 (33-81)

Stage	257 (76.5%)	253 (75.7%)
IIIc	77 (22.9%)	81 (24.3%)
IV	2 (0.6%)	0 (0%)

PFS	12 months	12 months

OS	29 months	30 months

#### Recurrent disease

Despite the activity of first-line chemotherapy, which gives response rates up to 80% in first line treatment, the majority of patients die of their recurrent disease [2]. Therefore, a large proportion of patients are candidates for second-line treatment. Platinum sensitivity, which is defined by a response to first-line platinum-based therapy, has been found to predict the response to subsequent retreatment with a platinum-containing regimen frequently used for salvage therapy.

In general, patients who progress or have stable disease during first-line treatment or who relapse within 1 month are considered to be 'platinum-refractory'. Patients who respond to primary treatment and relapse within 6 months are considered 'platinum-resistant', and patients who relapse more than 6 months after completion of initial therapy are characterized as 'platinum-sensitive' [11]. It is known that longer platinum free interval (PFI) increases the chances for a benefit by platinum re-challenge. This has been reported especially for PFI longer than 12 months. Patients who are relapsing 6-12 months following the end of their initial regimen may benefit less and are, usually classified as so-called 'partially sensitive' [12] (Table 4).

**Table 4:** Association of platinum sensitivity and PFI

Platinum sensitivity	resistant		sensitive	
	refractory	resistant	partially sensitive	sensitive

PFI	during/immediately after chemotherapy	< 6 months	6-12 months	> 12 months

Several randomized trials have been performed in platinum-sensitive patients. The ICON-4/OVAR 2.2 study compared the combination chemotherapy (platinum plus paclitaxel) to single chemotherapy (platinum alone) in 802 patients with 'platinum-sensitive' relapsed ovarian cancer. Results demonstrated that both survival and progression free survival were significantly longer in combination therapy compared to platinum alone [13].

The optimal treatment of patients with partially platinum-sensitive recurrent ovarian cancer is not clearly defined. Trabectedin, a marine-derived antineoplastic agent initially isolated from the tunicate *Ecteinascidia turbinate*, has recently been introduced to this setting of patients. This agent is currently produced synthetically and its mechanism of anti-cancer action is based on DNA minor-groove binding [14].

Patients with platinum refractory and resistant are good candidates for novel investigational approaches and studies of drug resistance. Single-agent therapy is considered the standard treatment in these patients. Low response rates are recorded in these patients with the use of topotecan, docetaxel, oral stoposide, pegylated liposomal doxorubicin (PLD), gemcitabine, ifosfamide and hexamethylmelamine. The pegylated liposomal doxorubicin (PLD), a new formulation of doxorubicin, compared with the conventional, assumes favorable pharmacokinetic properties such as a lower plasma concentration peak, lower clearance, smaller distribution volume, longer half-life and higher AUC, resulting in a different and more convenient toxicity and efficacy profile [15]. The efficacy of PLD has been clearly documented in recurrent ovarian cancer giving the rationale for its use also in the first-line setting. The MITO-2 (Multicenter Italian Trials in Ovarian cancer) phase III was designed to compare the combinations of carboplatin plus paclitaxel to an experimental arm with carboplatin plus PLD in first-line treatment of ovarian cancer patients. Results have been presented at ASCO 2010 showing that carboplatin plus PLD is not superior to carboplatin plus paclitaxel in terms of PFS; the median PFS was 19 and 16.8 months in the former and the latter arms, respectively. However, given the observed confidence interval and the different toxicity profile it has been proposed that carboplatin plus PLD could be considered an alternative to standard therapy [16].

Several randomized trials have been performed in platinum-sensitive patients. A multicenter phase III study, recently published, the Calypso study [12], has compared efficacy and safety of PLD-carboplatin and carboplatin-paclitaxel in 976 relapsed platinum-sensitive ovarian cancer patients. The trial showed superiority of the experimental arm in terms of PFS (11.3 months versus 9.4; HR = 0.821, 95% CI 0.72-0.94; *P * = 0.005). The safety profile of PLD-carboplatin appears remarkably different from that of carboplatin plus paclitaxel. The PLD-carboplatin combination was associated with a higher incidence of anemia and thrombocytopenia (rarely requiring transfusions) and a higher incidence of stomatitis and cutaneous toxicity (that were rarely severe, 14% of G1-2). Notably, however, the PLD-carboplatin combination was associated with a very low incidence of hair loss and neurotoxicity compared between the 2 arms was found in terms of response rate [16]. One interesting observation of this trial was in PLD-carboplatin arm compared to carboplatin-paclitaxel there was the reduction in the rate of hypersensitive reaction (grade > 2: 5.6% versus 18.8%) Therapeutic Strategies in Epithelial Ovarian Cancer and this is important information since hypersensitive reactions are reported in the general practice in patients treated with carboplatin up to 25%.

#### Treatment of clear cell type of EOC

Although clear cell type is categorized in Type I (indolent) ovarian cancer, it is known to show relatively strong resistance to carboplatin and paclitaxel regimen and thus poor prognosis compared to serous adenocarcinoma (SAC), especially in advanced stages. Previously Sugiyama et al. investigated clinical characteristics of clear cell carcinoma (CCC) of the ovary and showed that patients with CCC were significantly more likely to have FIGO Stage I disease than were patients with SAC (48.5% versus 16.6%). However, a high recurrence rate was noted in those patients with Stage IC CCC (37%) and the survival rates for those stage IC CCC patients were lower than those for patients with SAC. Also, the 3-year and 5-year survival rates for Stage III CCC patients were significantly lower compared with Stage III SAC patients [17].

Enomoto et al. demonstrated that clear cell or mucinous carcinoma histologic type did not respond to the carboplatin-paclitaxel combination chemotherapy (response rates 18%, 13%, respectively compared to 81% for serous adenocarcinoma and 89% for endometrioid adenocarcinoma) [18]. Considering those previous reports, alternative chemotherapy regimens or novel treatment for clear cell and mucinous carcinoma should be investigated.

Takakura et al. performed phase II trial of paclitaxel-carboplatin therapy (TC arm) versus irinotecan plus cisplatin therapy (CPT-P arm) as first-line chemotherapy for clear cell adenocarcinoma of the ovary [19]. PFS showed no significant difference between the 2 treatment groups. Because there were more patients with large residual disease in the CPT-P arm, they performed a subset analysis by removing those patients, and then compared the PFS with that of patients without residual disease less than 2 cm. The PFS tended to be longer in the CPT-P group, although the difference was not statistically significant. A phase III randomized trial of CPT-P arm versus TC arm undertaken by JGOG (Japanese Gynecologic Oncology Group) has closed and we are waiting for the results. According to a small retrospective in Japan, gemcitabine showed modest activity and is the most effective agent to clear cell adenocarcinoma of the ovary [20].

### History of chemotherapy regimens for EOC

Over the years, experts and research groups have explored different combinations of antitumor drugs in order to improve the prognosis of ovarian cancer (Table 5). In 1976, the report by Witshaw and Kroner on the efficacy of cisplatin in ovarian cancer produced the modern era of combination chemotherapy (platinum-based combination therapy).

**Table 5:** The history of chemotherapy regimens for ovarian cancer

Study	Chemotherapy	regimen Reference
GOG22	Melphalan < CA	Cancer 51:783, 1983

GOG47	CA < CAP	Cancer 57:1725, 1986

GOG52	CAP = CP	JCO 7:457, 1989

GOG111	CP < TP	NEJM 334:1, 1996

OV10	CP < TP	JNCI 92:699, 2000

GOG158	TP = TC	ASCO 1999; #1373, 1374

SCOTROC	TC = DC	ASCO 2002; #804

In the 1980s/early 1990 another turning point in the treatment of ovarian cancer was related to the discovery of paclitaxel, and active constituent of bark of the Pacific Yew tree, *Taxus brevifolia*. This agent acts by promoting microtubular assembly and stabilizes tubulin polymer formation and has a great deal of activity in epithelial ovarian cancer. Two randomized trials, the GOG 111 and the OV-10, comparing cisplatin/paclitaxel with cisplatin/cyclophosphamide, showed additional clinical benefit when cyclophosphamide was replaced by paclitaxel in the first-line setting [21-23].

Carboplatin, a cisplatin analogue is reported to have fewer marked side effects, especially such toxicities as nausea, renal toxicity, hearing loss, and neuromuscular toxicities than cisplatin. The carboplatin-paclitaxel combination is now considered an almost universal regimen in the management of epithelial ovarian cancer, and with a response rate of about 65%, PFS of 16-21 months and an OS of 32-57 months it is the standard arm in all the recent trials performed in this disease.

In the last two decades, some studies have been performed in order to improve the efficacy of first-line chemotherapy such as by delivering drugs in epithelial ovarian cancer through the intraperitoneal (IP) route.

GOG 172 phase III trial revealed a prolonged survival in the arm of intraperitoneal (IP) therapy compared to the arm of intravenous (IV) therapy (65.6 and 49.7 months respectively; *P *= 0.03). Also PFS was better in the IP-therapy arm than in the IV-therapy group (23.8 versus 18.3 months, *P *= 0.05) [24]. However, a significantly higher rate of both hematologic and non-hematologic toxicities, including catheter related complications was observed in the arm of IP chemotherapy in this study. In most countries the intravenous route of administration of chemotherapy is still preferred.

Some studies have investigated the possibility to substitute paclitaxel with other drugs in order to improve the efficacy of treatment and to reduce toxicities, in particular alopecia and neurotoxicity (Table 6) [25].

**Table 6:** Comparative investigations of the possibility to substitute paclitaxel with other drugs

Study	Treatment arms	FIGO stage	n	PFS (m)	OS(m)	*p*
SCOTROC-1		III-IV				0.71

	Carboplatin (AUC5)+Paclitaxel (175 mg/mq)		539	14.8	N.A	

	Carboplatin (AUC5)+Docetaxel (75 mg/mq)		538	15.0	N.A	

MITO-2		IC-IV				N.S.

	Carboplatin (AUC5) + Paclitaxel (175 mg/mq)		410	16.8	53.2	

	Carboplatin (AUC5) + Liposomal doxorubicin (30 mg/mq)		410	19.0	61.6	

N.A.: not accessed

N.S.: not significant

The first attempt to develop this strategy was performed with docetaxel, a semisynthetic taxane with pharmacologic and pharmacokinetic advantages, compared to paclitaxel. This approach was sustained by emerging evidences suggesting superiority over anthracyclines and paclitaxel in metastatic breast cancer [26,27].

In ovarian cancer, docetaxel demonstrated activity [28], both in paclitaxel-resistant patients [29], and in primary ovarian cancer, in association with carboplatin [30]. To further investigate these promising findings, the SCOTROC-1 phase III study was performed. 1077 patients with ovarian cancer were randomly assigned to receive carboplatin IV (AUC 5) plus either docetaxel at 75 mg/m2 (1-h intravenous infusion) or paclitaxel at 175 mg/m2 (3-h intravenous infusion) [31]. Contrary to the previous results from several preclinical studies, which suggested that docetaxel might be more beneficial to paclitaxel, this phase III study did not demonstrate a survival advantage for carboplatin plus docetaxel over carboplatin plus paclitaxel treatment.

Carboplatin plus paclitaxel combination was associated with higher neurotxicity than carboplatin plus docetaxel therapy. Conversely, treatment with carboplatin plus docetaxel was associated with statistically more events of G3-4 neutropenia (94% versus 84%, *P*<0.001) and neutropenic complications than other treatment, requiring the frequent use of G-CSF support. Based on these data docetaxel with carboplatin has been considered a possible alternative to carboplatin-paclitaxel treatment in patients at very high risk of neurotoxicity, but has not replaced carboplatin-paclitaxel as standard treatment.

According to a recent review article [32], gemcitabine was the most common drug used in clinical trials. Gemcitabine-based combination therapy showed an average response rate of 27.2%, and was the most common therapy among the group of regimens with above average response rate and progression-free survival.

### Novel treatment strategies of EOC

The larger expectation for improved prognosis in EOC is related to the use of the new biological agents. The deeper knowledge of ovarian cancer biology has led to the identification of multiple molecular targets, such as growth factor receptors, signal transduction pathways, cell cycle regulators, and angiogenic mechanisms. In this section, we overlook the major two molecular targeted agents applied to ovarian cancer treatment; anti-VEGF antibody bevacizumab and PARP inhibitor Olaparib.

#### Bevacizumab

One of the most investigated and promising molecular targeted drugs in ovarian cancer is bevacizumab, a monoclonal antibody directed against VEGF. VEGF expression is higher in ovarian cancer tumors than in normal ovarian tissue or benign ovarian tumors, and increasing VEGF expression in either cytosolic fractions derived from ovarian cancer tumors or serum VEGF levels in preoperative serum is considered to be associated with advanced stage and worse survival.

In order to inhibit the VEGF pathway, there are two primary strategies: (1) inhibition of the VEGF ligand with antibodies or soluble receptors and (2) inhibition of the VEGF receptor (VEGFR) with tyrosine kinase inhibitors (TKIs), or receptor antibodies. Of the VEGF targeting therapies, the most experience has been with a monoclonal antibody that binds the VEGF ligand, known as bevacizumab (Avastin). Bevacizumab is a 149-kDa recombinant humanized monoclonal IgG1 anti-VEGF antibody. It has been FDA-1 approved for the treatment of metastatic colorectal, breast, and non-small cell lung cancer and shows promise in the treatment of ovarian cancer. Several phase II studies have shown that bevacizumab is active in recurrent ovarian cancer [33,34].

Two phase III trials (GOG218, ICON 7) have recently evaluated the role of bevacizumab in first-line chemotherapy as an adjunct to carboplatin and paclitaxel. The GOG 218 is a multicenter, placebo-controlled trial with the primary end point to determine whether the addition of bevacizumab (15 mg/kg every 21 days) to standard chemotherapy is able to prolong PFS after primary cytoreductive surgery. Recently bevacizumab plus chemotherapy (carboplatin-paclitaxel) and bevacizumab maintenance was demonstrated to be able to prolong PFS of about 4 months (10.3 months versus 14.1 months) compared to carboplatin-paclitaxel alone [35]. Another multicenter trial is the ICON 7, an open label, two-arm trial, enrolling patients with high risk or advanced (stage I-IV) epithelial ovarian cancer to receive carboplatin plus paclitaxel or carboplatin-paclitaxel plus bevacizumab given concurrently and as maintenance up to 18 cycles. The bevacizumab used in this trial was half of that given in the GOG 218 study. This trial also showed that the addition of bevacizumab is able to prolong PFS compared to standard carboplatin-paclitaxel [36].

Another study, OCEANS trial, showed that addition of bevacizumab prolonged PFS in platinum-sensitive recurrent ovarian carcinoma cases [37].

#### PARP inhibitor, olaparib

The poly (ADP-ribose) polymerases (PARPs) are a large family of multifunctional enzymes [38]. PARP-1, the most abundant isoform, plays a key role in the repair of DNA single-strand breaks through the repair of base excisions. The inhibition of PARPs leads to the accumulation of DNA single-strand breaks, which causes DNA double-strand breaks at replication forks. These double-strand breaks are repaired in normal cells mainly by the error-free homologous recombination double-stranded DNA repair pathway, in which essential components are the tumor-suppressor proteins BRCA1 and BRCA2. In the absent of either BRCA1 or BRCA2, these lesions are not repaired, which results in cell cycle arrest and cell death, although there is an alternate pathway to non-homologous end-joining for DBS repair [39].

Women with inherited mutations in BRCA1 on chromosome 17q21 or BRCA2 on chromosome 13q31 are at significantly higher risk of developing breast and ovarian cancer than women in the control population. The lifetime risks of ovarian cancer are 54% for BRCA1 and 23% for BRCA2 mutation carriers [40]. Inherited mutations in those genes are found in 5-10% of all ovarian cancer patients. However, over 50% of high-grade serous or undifferentiated carcinomas (Type II ovarian cancer) showed loss of BRCA function, either by genetic or epigenetic events, which resulted in HR DNA repair defects [41].

The discovery of epigenetic mechanism of BRCA1/2 germinal mutation and the association of this mutation with ovarian cancer in 5-10% of the cases, led to the therapeutic concept of "synthetic lethality" [42]. In fact, in patients carriers BRCA mutation, PARP inhibition results in unrepaired DNA single-strand and double strand breaks and so cell death [43].

Fong et al. administered to fifty patients, the majority of which were platinum refractory, the PARP inhibitor olaparib with a favorable safety profile and a high response rate, in particular in patients with BRCA mutation. In patients with platinum-resistant and even platinum-refractory disease the response rate (of PARP inhibitor, olaparib) was of 41.7% and 15.4%, respectively [44]. Olaparib (AZD2281) was tested in BRCA-mutated patients with ovarian, primary peritoneal, and fallopian tube cancer. In the study, 20 patients (40%) responded to the therapy. Currently, randomized trials of olaparib and other PARP inhibitors in patients with ovarian cancer are underway.

## Conclusion

Maximal surgical cytoreduction followed by systemic taxane and platinum-based chemotherapy is the standard treatment for patients with ovarian cancer. Molecular targeting therapy may improve the prognosis of them.

## Abbreviations

CA: Cyclophosphamide + Adriamycin; CAP: Cyclophosphamide + Adriamycin + Cisplatin; CP: Cyclophosphamide+ Cisplatin; TP: Paclitaxel + Cisplatin; TC: Paclitaxel + Carboplatin; DC: Docetaxel + Carboplatin

## Competing interests

The authors declare that they have no competing interests.

## Authors' contributions

Dr. K wrote the manuscript, and Dr. E, Dr. U and Dr. N approved it. All authors read and approved the final manuscript.
